# Development of a Novel Antibacterial Peptide, PAM-5, via Combination of Phage Display Selection and Computer-Assisted Modification

**DOI:** 10.3390/biom13030466

**Published:** 2023-03-02

**Authors:** Hawk Leong Yuen, Szn Yi Chan, Yi En Ding, Suxing Lim, Gim Cheong Tan, Chiew Ling Kho

**Affiliations:** 1 Department of Allied Health Sciences, Faculty of Science, Universiti Tunku Abdul Rahman, Kampar 31900, Perak, Malaysia; 2 Department of Biological Science, Faculty of Science, Universiti Tunku Abdul Rahman, Kampar 31900, Perak, Malaysia

**Keywords:** antibacterial peptide, PAM-5, phage display, peptide modification, antibacterial activity

## Abstract

Antibacterial peptides (ABPs) have been proposed as potential candidates for alternative antibacterial agents due to the extensive dissemination of antibiotic resistance. However, ABP isolation from natural resources can be tedious without consistent yield. Moreover, many natural ABPs are not developed for clinical application due to potential toxicity to mammalian cells. Therefore, the objective of this study was to develop a potent ABP with minimal toxicity via phage display selection followed by computer-assisted modification. Briefly, a 12-mer phage-displayed peptide library was used to isolate peptides that bound to the cell surface of *Pseudomonas aeruginosa* with high affinity. The affinity-selected peptide with the highest selection frequency was modified to PAM-5 (KWKWRPLKRKLVLRM) with enhanced antibacterial features by using an online peptide database. Using in vitro microbroth dilution assay, PAM-5 was shown to be active against a panel of Gram-negative bacteria and selected Gram-positive bacteria. Interestingly, the peptide was stable in human plasma by exhibiting a similar bactericidal effect via ex vivo assay. Scanning electron microscopy and SYTOX Green uptake assay revealed that PAM-5 was able to cause membrane disruption and permeabilization of the bacteria. Additionally, the peptide was also able to bind to bacterial DNA as demonstrated by gel retardation assay. In the time-kill assay, PAM-5 was shown to kill the bacteria rapidly in 10 min. More importantly, PAM-5 was non-cytotoxic to Vero cells and non-haemolytic to human erythrocytes at all concentrations tested for the antibacterial assays. Thus, this study showed that the combination of phage display screening and computer-assisted modification could be used to develop potent novel ABPs, and PAM-5 derived from these approaches is worth to be further elucidated for its potential clinical use.

## 1. Introduction

Despite the global collaborative efforts to develop prophylactic and therapeutic agents against the recently emerging SARS-CoV2, continuous research and development for novel alternative antibacterial agents to address the pre-existing issue of antibiotic resistance should not be neglected. In fact, many complicated bacterial infections which are secondary to viral infections such as COVID-19 [1,2,3] remain as a serious problem that necessitates more effective antibacterial agents to reduce morbidity and mortality rates. However, most of the commonly used antibiotics in clinical settings, including the final resort options such as carbapenems, have been compromised by bacterial resistance [4,5]. Apart from the extrinsic factors that induce antibiotic resistance such as abuse and patients’ non-compliance in consumption, the intrinsic limitations of antibiotics such as slow killing kinetics [6,7,8] and site restriction of antibiotic action [9,10,11] may also compromise their efficacy and potency towards their target bacteria. These shortcomings may confer the bacteria with sufficient time and opportunity to acquire mutation-mediated resistance to the drugs. Therefore, it is an urgent need to develop novel antibacterial agents with rapid killing kinetics and non-ligand-specific action which could circumvent the bacterial resistance mechanisms.

Over the decades, antibacterial peptides (ABPs) have been extensively studied for their potential application to complement the currently used conventional antibiotics against bacterial infections. Numerous potent ABPs have been isolated from natural sources [12,13,14,15,16]. However, the processes of isolating and purifying these natural ABPs are usually tedious and laborious without ensuring high yield [17,18], and many of these peptides were found cytotoxic or haemolytic to human cells [19,20,21]. These major drawbacks have prompted alternative approaches in ABP development for better therapeutic index [22,23].

One of these methods is phage display selection of peptides that bind to bacterial ligand/s with high affinity. This method offers a convenient and high-throughput screening strategy to identify peptides that selectively interact with bacterial surface ligands from a phage-displayed peptide library. After several rounds of biopanning the library against a target bacterium, peptides that consistently bound to the bacterial surface ligand/s could be isolated and identified through sequencing of the phage DNA that encode the peptides. This approach has been used in several studies to produce novel ABPs with potent antibacterial effects [24,25,26,27]. Nevertheless, although phage display selection may isolate peptides that selectively bind to bacterial ligands, it does not ensure the optimum cationicity, hydrophobicity as well as peptide length that are required to interact with bacterial membranes [24,25,26,27].

Therefore, in this study, we aimed to develop an ABP with potent bacteriostatic/bactericidal effect but low toxicity by combining phage display selection and computer-assisted peptide modification. The former approach enabled us to isolate several peptide candidates that bound to target bacterial surface ligands, while the latter aimed to adjust the peptide length, cationicity and hydrophobicity for improvement of antibacterial potency and reduction of peptide toxicity. The resulting peptide was evaluated for its antibacterial efficacy, peptide stability, killing kinetics, mechanisms of action and toxicity.

## 2. Materials and Methods

### 2.1. Bacterial Strains and Growth Conditions

The species and strains of bacteria used in this study encompassed ATCC reference strains which were provided by Department of Allied Health Sciences, Faculty of Science, Universiti Tunku Abdul Rahman, as well as drug- or multidrug-resistant clinical strains which were provided by Pathology Laboratory, Gleneagles Hospital, Penang. Stock cultures of all these bacteria were prepared in Luria Bertani (LB) broth (Merck Millipore) with 25% glycerol and stored at −80 °C. Bacterial cultures were prepared from the glycerol stock by inoculating on Muller Hinton (MH) agar plates (Merck Millipore) and stored at 4 °C for a maximum of seven days.

### 2.2. Phage Display Selection of Peptides Binding to P. aeruginosa

Peptides that bind to *P. aeruginosa* were selected via biopanning of a 12-mer random phage-displayed peptide library against the bacterial whole cells according to the protocol as described by the manufacturer (New England Biolabs, Ipswich, MA, USA) [28]. For the first round of biopanning, *P. aeruginosa* ATCC 27853 resuspended in 2 mL of PBS (pH 7.4) was added with 10 µL of the phage library diluted in 100 µL of TBST, followed by incubation at room temperature for 60 min with gentle agitation. Unbound phages were removed by a series of washing with TBST followed by centrifugation. Bound phages were eluted with 200 µL of elution buffer [0.2 M Glycine-HCl (pH 2.2), 1 mg/mL BSA] for 10 min at room temperature, followed by neutralization of the eluted phages with 30 µL of 1 M Tris-HCl (pH 9.1). A titer of the eluted phages was determined by plating an aliquot of the phages on LB IPTG/Xgal agar, while the rest of the phages were amplified in *Escherichia coli* ER2738. Amplified phages were purified with polyethylene-glycol precipitation, followed by resuspension of the phage pellet in TBS. The titer of the amplified phages was determined before proceeding to the subsequent round of biopanning. A total of four rounds of biopanning were carried out to enrich the clones of phage-displayed peptides binding to *P. aeruginosa*, with the titers of input phages (phages in TBS after amplification) and output phages (phages in elution buffer) being determined each round. After the final round of biopanning, individual eluted phages on the titer plate were randomly selected for amplification in *E. coli* ER2738 to be used in phage-ELISA and phage genomic DNA extraction.

### 2.3. Phage-ELISA Screening for Phage Peptides Binding to P. aeruginosa

Wells of a 96 well-microtiter plate (Greiner CELLSTAR^®^, Germany) were coated with 100 µL of *P. aeruginosa* ATCC 27853 in PBS at a titer of 1.2 × 10^5^ CFU/mL. After overnight incubation at 4 °C, bacteria in the wells were fixed with ethanol for 10 min followed by airdrying. The wells were blocked with 200 µL of blocking buffer [0.1 M NaHCO_3_ (pH 8.6), 5 mg/mL BSA, 0.02% NaN_3_] followed by 1 h incubation at 4 °C. After rinsing the wells with PBS (pH 7.4), 150 µL of the individual amplified phage peptides at the titer of 10^11^ PFU/mL was added to the bacterial-coated wells (each clone for a well), and the plate was incubated on a rotary shaker for 1 h at room temperature. After washing the plate with TBST (TBS pH 7.4, 0.5% Tween 20) for 5 times, the wells were filled with 200 µL of 1:1000-diluted HRP-linked anti-M13 mAb and incubated for 1 h at room temperature. After 6 times washing with 0.5% TBST, the wells were added with 200 µL of 2,2′-azino-bis (3-ethylbenzothiazoline-6-sulfonic acid) diammonium salt (ABTS) (Sigma-Aldrich, St. Louis, MO, USA) in 50 mM of sodium citrate (pH 4.0) and 30% H_2_O_2_. The color development in the wells was recorded by FLUOstar Omega microplate reader (BMG LabTech) at the wavelength of 405 nm.

### 2.4. Phage Genomic DNA Sequencing and Peptide Determination

Phage clones displaying peptides with high binding affinity to *P. aeruginosa* in the phage-ELISA assay were selected for genomic DNA extraction and sequencing according to the protocol as described by the manufacturer (New England Biolabs, MA). The complementary sequence of the oligonucleotide inserts that encoded for the displayed peptides were determined and deduced into peptide sequences by using ExPASy DNA translate tool (http://web.expacy.org/translate/, accessed on 18 January 2017). The physiochemical properties of the peptides were analyzed by the Antimicrobial Peptide Calculator and Predictor (http://aps.unmc.edu/AP/main.php, accessed on 22 January 2017). The peptides were aligned using Multiple Sequence Alignment (MSA) from ClustalW2 (http://ebi.ac.uk/Tools/msa/emboss_cons/, accessed on 3 February 2017) to determine the frequency of selection for a particular peptide sequence, as well as to identify any consensus motif/s between the selected peptides.

### 2.5. Peptide Modification and Synthesis

Pa4 (KWHWKDKNALRM), the 12-mer peptide with the highest selection frequency from the biopanning, was rationally modified by using Antimicrobial Peptide Calculator and Predictor (http://aps.unmc.edu/AP/prediction/prediction_maim.php, accessed on 15 April 2017) and Antimicrobial Peptide Designer (http://aps.unmc.edu/AP/design/design_improve.php, accessed on 15 April 2017) to enhance the peptide length, cationicity and hydrophobicity. Firstly, the peptide was elongated to 15-mer by adding arginine (R), proline (P) and leucine (L) between the tryptophan (W) and leucine (L) at the fourth and fifth amino acid of Pa4, respectively. Then, the less cationic or anionic residues in the peptide such as histidine (H) and aspartic acid (D) were substituted with cationic lysine (K) and arginine (R), while the less hydrophobic residues asparagine (N) and alanine (A) were replaced by leucine (L) and valine (V). The newly modified peptide, namely PAM-5 (KWKWRPLKRKLVLRM), was chemically synthesized by standard solid-phase methodology (BioBasic Co., Markham, ON, CA). The purity (>95%) and molecular weight of the peptide were determined by high performance liquid chromatography (HPLC) and mass spectrometry, respectively.

### 2.6. Antibacterial Effect of PAM-5 In Vitro

The antibacterial effect of PAM-5 against selected bacteria of ATCC reference strains and clinical drug-resistant strains was determined using broth microdilution assay as previously described [29,30], with slight modification. Briefly, using the corresponding broth media for respective bacterial growth, the bacteria were grown to their mid-log phase (OD_605_~0.5). After adjusting to an inoculum titer of 10^4^–10^5^ CFU/mL with PBS (pH 7.4), the bacteria were treated with PAM-5 at concentrations ranging from 4 µg/mL to 256 µg/mL in the wells of a 96-well polypropylene microtiter plate. Untreated bacteria in PBS (pH 7.4) were set up as negative control, while bacteria treated with polymyxin B served as the positive control. After 24 h of incubation at 37 °C, the minimum inhibitory concentration (MIC) of PAM-5 was determined as the lowest peptide concentration that inhibited visible bacterial growth in the well. After MIC determination, dilutions of the treated bacterial suspension were plated on Mueller Hinton (MH) agar for colony-forming unit (CFU) counting. Minimum bactericidal concentration (MBC) of PAM-5 was defined as the lowest peptide concentration that killed ≥ 99.9% of the bacterial inoculum, as recommended by CLSI [29].

### 2.7. Antibacterial Effect of PAM-5 in Human Plasma

The stability of PAM-5 in human plasma was assessed by ex vivo microbroth dilution assay. *P. aeruginosa* ATCC 27853, which was used as the representative bacteria in this study, was grown to its mid log phase (OD_605_~0.5) and diluted to 10^5^ CFU/mL with fresh human plasma provided by the Pathology Laboratory, Gleneagles Hospital, Penang. Plasma-suspended bacteria were treated with PAM-5 at increasing concentrations from 4 µg/mL to 256 µg/mL. Both polymyxin B-treated and untreated bacteria in PBS were set up as the positive and negative control, respectively. After overnight incubation at 37 °C, the MBC of PAM-5 was determined as described earlier.

### 2.8. Time-Kill Assay

Bacterial killing kinetics by PAM-5 was assessed by colony counting of the peptide-treated bacteria after certain time-points. The assay was performed on *P. aeruginosa* ATCC 27853 and *E. coli* ATCC 35218 as representative strains for the selected Gram-negative bacteria in this study. Briefly, bacterial suspension in PBS (pH 7.4) at the inoculum titer of ~10^3^ CFU/mL was treated with PAM-5 at 2× MBC against the bacteria. Simultaneously, two sets of the same bacteria were also set up for treatment with gentamycin and polymyxin B at their 2× MBCs as well. Untreated bacteria in PBS were set up as the negative control. Upon treatment, aliquots of 20 µL of the treated and non-treated bacteria were aspirated at different time points (0, 10, 20, 30, 40, 50 and 60 min) and inoculated on MH agar plates via spread plate method. After overnight incubation at 37 °C, the number of colonies on the plates was counted and viable bacteria were expressed as log_10_ CFU/mL.

### 2.9. Scanning Electron Microscopy (SEM) on PAM-5-Treated Bacteria

As described previously [31,32], SEM was performed to screen for any morphological changes to PAM-5-treated *P. aeruginosa* and *E. coli*. Briefly, 100 µL of bacteria at the titer of ~10^7^ CFU/mL was treated with 100 µL of PAM-5 at the final concentration of 64 µg/mL. Non-treated bacteria in PBS were set up as the negative control. After incubation for 3 h at 37 °C, the bacteria were centrifuged at 6000× *g* for 6 min. The bacterial pellet was then washed 3 times with PBS (pH 7.4) and fixed with 3% (*v*/*v*) glutaraldehyde in 0.1 M of PBS at 4 °C for 18–24 h. After removing the glutaraldehyde via centrifugation (4000× *g*, 5 min), the bacteria were washed 2 times with 0.1 M PBS and step-dehydrated with ethanol at increasing concentrations of 25%, 50%, 75%, 95% and 100% (*v*/*v*). After freeze-drying the bacterial pellet for 18 h, the cells were subjected to gold-coating and examined by SEM (JSM-7610F, Japan).

### 2.10. Membrane Permeabilization by PAM-5 via SYTOX Green Uptake Assay

The ability of PAM-5 to permeabilize bacterial cytoplasmic membrane was screened through SYTOX Green uptake assay. *P. aeruginosa* ATCC 27853 at the titer of ~ 10^5^ CFU/mL was treated with PAM-5 at increasing concentrations from 2 µg/mL to 256 µg/mL in a flat- bottom, black opaque, 96-µClear Chimney well microtiter plate (Greiner Bio-One, Frickenhausen, Germany). Concurrently, polymyxin B-treated and non-treated bacteria in PBS were set up as the positive and negative control, respectively. After 1 h of incubation at 37 °C, each well was added with 50 µL of 1 µM SYTOX Green (Invitrogen, Carlsbad, CA, USA). After 15 min of incubation in the dark, the fluorescent signals from the wells were measured by a microplate reader (Tecan Infinite M200), in which the excitation and emission wavelengths were set at 485 nm and 520 nm, respectively.

### 2.11. DNA Retardation Assay

The ability of PAM-5 to bind bacterial nucleic acids was screened by electrophoretic mobility shift assay (EMSA). Genomic DNAs were extracted and purified from *P. aeruginosa* ATCC 27853 and *E. coli* ATCC 35218 using standard protocols as described by Green and Sambrook (2017) [33]. Plasmid DNA pBR322 was purchased from Thermo Fisher Scientific (USA). These nucleic acids were diluted to 100 ng/µL by Tris-EDTA (TE) buffer [10 nM Tris (pH 8.0), 1 mM EDTA], followed by incubation with PAM-5 at increasing concentrations from 4 µg/mL to 256 µg/mL. Untreated nucleic acid was added with TE buffer (pH 8.0), which served as the negative control. The mixtures were incubated for 1 h at room temperature, and then subjected to electrophoresis on a 1% agarose gel in TBE buffer. The gel was stained with ethidium bromide and the DNA bands were visualized under a Syngene Gel Documentation system (UK).

### 2.12. Cell Cytotoxicity Assay

The toxicity of PAM-5 was screened on Vero cell line using PrestoBlue^TM^ Cell Viability assay according to the manufacturer’s protocol (Invitrogen, Carlsbad, CA, USA). The cells were cultured in Dulbecco’s Modified Eagle’s Medium (DMEM) supplemented with 10% (*v*/*v*) fetal bovine serum (FBS), 2 mM of glutamine and 1% (*v*/*v*) of penicillin-streptoycin solution, followed by seeding the cells in the wells of a white-opaque, flat-bottom 96-well-microtiter plate (Greiner-Bio, Germany), with approximately 6 × 10^4^ cells/well. The cells were treated with PAM-5 and polymyxin B at various concentrations ranging from 2 µg/mL to 256 µg/mL. Cells added with 10% (*v*/*v*) DMSO and PBS (pH 7.4) were set up as the positive and negative control, respectively. After overnight incubation at 37 °C in a 5% CO_2_ humidified condition, 20 µL of PrestoBlue^TM^ reagent was added to the wells. The plate was further incubated for another 24 h at 37 °C in 5% CO_2_. Finally, the relative fluorescent units (RFUs) in the wells were measured by a microplate reader (BMG Labtech) with excitation and emission wavelength at 544 nm and 620 nm, respectively.

### 2.13. Haemolytic Effect of PAM-5

PAM-5 was also tested for its haemolytic effect towards human red blood cells (hRBCs) using in vitro haemolytic assay. RBC sample was kindly provided by a healthy volunteer with ethical approval from the university. After 3 times of washing with PBS (pH 7.4), 10% (*v*/*v*) of the RBC suspension was treated with PAM-5 at increasing concentrations from 2 µg/mL to 256 µg/mL. RBCs added with 0.1% Triton-X and untreated RBCs in PBS (pH 7.4) were used as the positive and negative control, respectively. The treated and non-treated RBCs were then incubated for 1 h at room temperature, followed by centrifugation at 4000× *g* for 5 min at room temperature. Haemolysis of RBCs was monitored by measuring the absorbance of the supernatant after the centrifugation. The percentage of haemolysis was calculated by using the equation below:

Percentage of haemolysis (%) = (A_s_ − A_n_/A_p_ − A_n_) × 100%

where A**_s_** represents the absorbance of each treatment group, A**_p_** represents the average absorbance of the positive control group for haemolysis, and A**_n_** represents the average absorbance of the negative control group.

### 2.14. Statistical Analysis

The assays for phage-ELISA, in vitro and ex vivo antibacterial effect, kinetic killing, membrane permeabilization, DNA retardation, in vitro cell cytotoxicity and haemolytic effect as described above were triplicated for data reproducibility The data were recorded as mean + SD. Statistical analysis to determine the significant differences on data sets was performed using one-way ANOVA, where the differences were considered significant when *p* < 0.05.

## 3. Results

### 3.1. Affinity-Selected Peptides Binding to P. aeruginosa

Using the 12-mer random phage-displayed peptide library, 30 clones of phage-displayed peptides that bound to whole cells of *P. aeruginosa* were randomly isolated after four consecutive rounds of biopanning. Upon phage-ELISA screening, 15 clones of peptides with the highest binding affinity to the bacterium were selected for DNA extraction and sequencing. Peptide deducing from the DNAs revealed that these 15 peptides could be segregated into seven groups as tabulated in Table 1. Among these groups, Pa4, with the peptide sequence KWHWKDKNALRM, demonstrated the highest selection frequency from the biopanning (5/15). On the other hand, Pa1, which represented the second-highest selected group (3/15), encoded a peptide sequence GPVNKSSTILRM that shared the same motif consisting of leucine-arginine-methionine (LRM) with Pa4 at the carboxyl terminus. Two other groups, designated as Pa3 (GLHTSATNLYLH) and Pa5 (GSLRPGTTNALV), also possessed a leucine (L) residue near the carboxyl terminal of the peptides. The remaining peptide groups each encoded unique peptide sequences.

### 3.2. Peptide Modification

As Pa4 represented the most selected peptide from the biopanning, along with the consensus motif “LRM” at the same position of the two most frequently selected peptide groups (Pa1 and Pa4), it was chosen for further antibacterial evaluation. However, based on the online prediction of antimicrobial peptide in the Antimicrobial Peptide Database (https://aps.unmc.edu/prediction/predict, accessed on 15 March 2017), Pa4 may not be sufficient to fulfil the criteria of an ideal antibacterial peptide as it only exhibited moderate cationicity and hydrophobicity of +3 and 41%, respectively. Therefore, the original peptide sequence of Pa4 (KWHWKDKNALRM) was modified to enhance its cationicity and hydrophobicity as described. The newly modified peptide, PAM-5, with the sequence of KWKWRPLKRKLVLRM, had a greater cationic strength and hydrophobicity of +7 and 47%, respectively. With reference to the same online prediction, PAM-5 was shown to possess some features of antibacterial peptides. However, the peptide was not significantly matched to any pre-existing antimicrobial peptides documented in the database.

### 3.3. Antibacterial Effect of PAM-5

The newly modified peptide was tested for its in vitro antibacterial effect on *P. aeruginosa* ATCC 27853, the target bacterium which was used to select for its parental peptide. Besides, the peptide was also screened for its antibacterial spectrum on a panel of other bacteria in which their corresponding antibiotic susceptibility profiles were tabulated in Table 2. The online prediction on the antibacterial potency of PAM-5 as mentioned above was in concordance to the findings from the in vitro antibacterial assay. As shown in Table 2, PAM-5 was shown to be bactericidal towards *P. aeruginosa* ATCC 27853 at the MBC of 8 µg/mL. Moreover, PAM-5 was also active against other reference strain of Gram-negative bacteria such as *E. coli* ATCC 25922 (MBC = 8 µg/mL), *A. baumannii* ATCC 19606 (8 µg/mL) and *K. pneumoniae* ATCC 138833 (32 µg/mL). Similarly, PAM-5 was also potent against several multidrug-resistant clinical isolates of these Gram-negative bacterial counterparts at MBCs that ranged from 4 µg/mL to 32 µg/mL. Interestingly, this panel included a carbapenem-resistant *K. pneumoniae* (1208398), which is generally regarded as a superbug. However, PAM-5 failed to inhibit or kill *S. marcescens* at all tested concentrations. In terms of Gram-positive bacteria, except for *S. anginosus* (MBC 4 µg/mL), PAM-5 could only eliminate *S. pyogenes* and *S. aureus* at high concentrations (MBCs: 64 -128 µg/mL), but was unable to kill *E. faecalis* at all tested concentrations.

### 3.4. Stability of PAM-5 in Human Plasma

The stability of PAM-5 in human plasma was accessed with an ex vivo antibacterial assay against *P. aeruginosa* ATCC27853 as described in Materials and Methods, and the relative MBCs between the in vitro and ex vivo assays were compared. The findings showed that PAM-5 was able to kill the bacterium completely at 16 µg/mL (data not shown), which was 2-fold higher than the MBC for the in vitro assay.

### 3.5. Killing Kinetics of PAM-5

**Figure 1:**
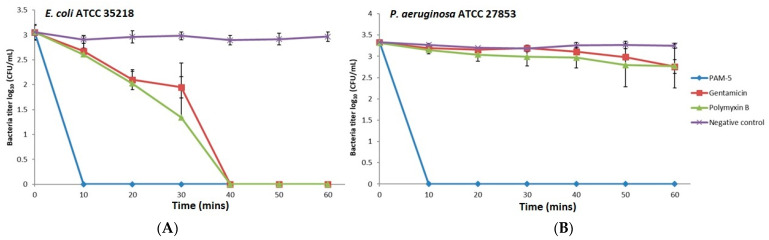
Killing kinetics of PAM-5 on (**A**) *E. coli* ATCC 35218 and (**B**) *P. aeruginosa* ATCC 27853. The data were presented as means (+ SD) of three independent assays.

The potent bactericidal effects of PAM-5 on the selected Gram-negative bacteria as reported above were determined after overnight treatment of the bacteria with the peptide. However, this did not provide insights on how fast the peptide achieved complete killing of the bacteria. To address this, we studied the time-killing kinetics of PAM-5 on *P. aeruginosa* ATCC 27853 and *E. coli* ATCC 35218 for a period of one hour and compared it to the killing kinetics of polymyxin B and gentamycin. Clearly demonstrated from Figure 1, at 2 × MBC (16 µg/mL), PAM-5 killed both the bacteria completely in 10 min upon peptide treatment. In contrast, a longer duration (40 min) was needed by polymyxin B and gentamycin to eliminate *E. coli* (Figure 1A), while no remarkable bacterial titer reduction was seen for *P. aeruginosa* within this period after treatment with both the antibiotics (Figure 1B).

### 3.6. SEM Examination on Morphology of PAM-5-Treated Bacteria

**Figure 2:**
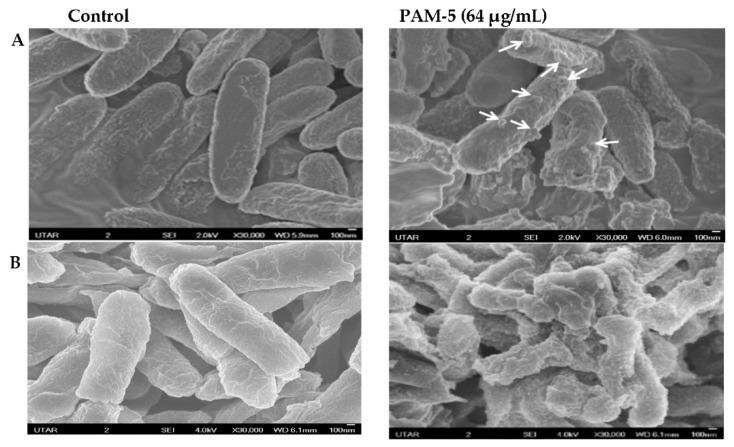
SEM micrographs of peptide-treated and untreated *P. aeruginosa* ATCC 27853 (**A**) and *E. coli* ATCC 25922 (**B**) observed under 30,000× magnification. Smoother surfaces were apparent for untreated bacteria (control). In contrast, surface roughening and corrugation are clearly seen from the bacteria treated with 64 µg/mL of PAM-5. Small protuberant structures can be seen on the surface of PAM-5-treated bacteria (indicated by arrows).

The morphology of PAM-5-treated *P. aeruginosa* and *E. coli* was observed under SEM for any structural difference as compared to the untreated bacteria. As shown in Figure 2, untreated *P. aeruginosa* (A) and *E. coli* (B) which served as the negative controls were morphologically and structurally intact with a smooth surface. In contrast, PAM-5-treated bacteria displayed higher degree of surface corrugation and roughening, along with small protuberant structures or blebbing that were present extensively throughout the entire bacterial outer surface.

### 3.7. Cytoplasmic Membrane Permeabilization by PAM-5

The findings on the structural disruption by PAM-5 as observed in SEM raised a speculation that the peptide may cause permeabilization of the bacterial inner plasma membrane. Therefore, we used SYTOX Green, a membrane-impermeable, DNA-binding

**Figure 3:**
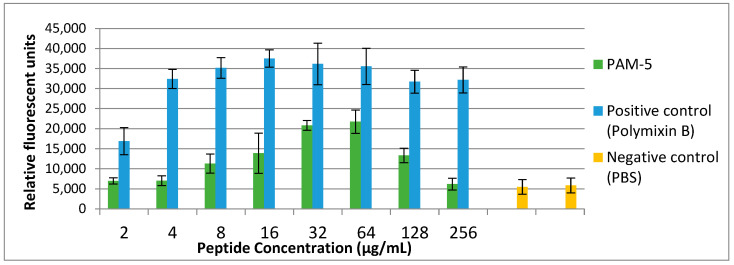
Bacterial cytoplasmic membrane permeabilization by different peptide concentrations as determined by SYTOX^®^ Green uptake assay. *P. aeruginosa* with the titer of 10^6^ CFU/mL was treated with increasing concentrations of PAM-5 ranging from 2 µg/mL to 256 µg/mL. Bacteria treated with polymyxin B of the same range of concentrations served as the positive control, while untreated bacteria served as the negative control. The data were presented as means (+SD) of three independent assays.

Fluorescent dye to address this on *P. aeruginosa* ATCC 27853. As demonstrated in Figure 3, low fluorescence was consistently observed from the untreated bacteria (negative control). Conversely, polymyxin B-treated bacteria, which served as the positive control, demonstrated increasing SYTOX Green fluorescent signals in a dose-dependent manner. Correspondingly, increasing fluorescent signals were also seen for bacteria treated with PAM-5 at concentrations ranging from 2 µg/mL to 64 µg/mL. However, the fluorescent intensity was seen declining from the bacteria treated with 128 µg/mL and 256 µg/mL of PAM-5. This unexpected pattern of fluorescent emission was consistent throughout three independent assays.

### 3.8. PAM-5 Binding to Bacterial Genomic DNA and Plasmid DNA

**Figure 4:**
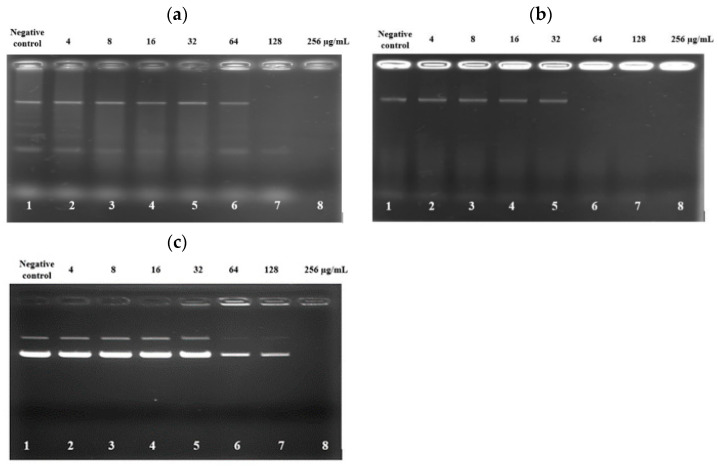
Gel retardation assay on the binding of PAM-5 to genomic DNA extracted from (**a**) *P. aeruginosa* ATCC 27853; (**b**) *E. coli* ATCC 35218; and (**c**) pBR322 plasmid DNA from *E. coli*. Lane 1: untreated DNA; lanes 2 to 8: DNAs treated with increasing concentrations of PAM-5 ranging from 4 µg/mL to 256 µg/mL. The assays were triplicated with consistent similar pattern of migrations.

The deceasing trend of SYTOX Green fluorescent emission from the bacteria treated with high concentrations of PAM-5 as reported above raised the speculation that the peptide may bind to bacterial DNA at these concentrations. To verify this, an electrophoretic gel mobility shift assay was carried out to screen for the ability of PAM-5 to bind genomic DNA from *P. aeruginosa* and *E. coli*. As demonstrated in Figure 4a, at high concentrations of 128 µg/mL and 256 µg/mL, PAM-5 was able to retard electrophoretic mobility of DNAs from *P. aeruginosa* (Lane 7 and 8). The strength of retardation was even greater towards the DNA from *E. coli*, as no sign of migration was observed for DNAs treated with the peptide at 64 µg/mL onwards (Figure 4b, Lane 6–8). On the other hand, similar inhibition of electrophoretic mobility was also seen for plasmid DNA pBR322. At the concentrations of 64 µg/mL and 128 µg/mL, the intensities of the bands that represented the supercoiled and relaxed forms of the plasmid were greatly reduced as compared to the untreated plasmid (Figure 4c, Lane 6 and 7). At 256 µg/mL (Lane 8), migration of the plasmid was totally retarded.

### 3.9. Toxicity Effects of PAM-5 on Vero Cells

**Figure 5:**
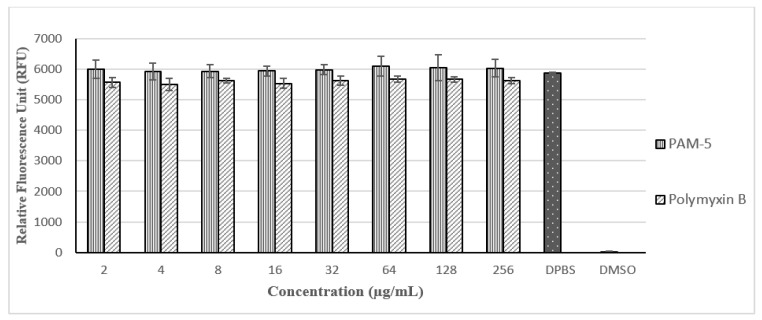
Relative fluorescence units (RFUs) produced by Vero cells after different treatments based on PrestoBlue assay. RFUs produced by cells treated with PAM-5 and polymyxin B were relatively similar to cells in DPBS, which served as the negative control for toxicity. Cells treated with 50% DMSO, which served as the positive control for toxicity, generated extremely low RFU.

With reference to Figure 5, PAM-5 was not toxic to Vero cells at all the tested concentrations (2 µg/mL to 256 µg/mL) as there were no significant difference in the RFUs between the peptide-treated and untreated cells (*p* > 0.05). Moreover, no trend of decreasing RFU due to increasing concentration of PAM-5 from 2 µg/mL to 256 µg/mL was observed in the cells, suggesting the viability of the cells was not affected by the peptide in a dose-dependent manner at these concentrations. In contrast, these signals were significantly much higher than from the DMSO-treated cells, which served as the positive control for toxicity (*p* < 0.05). On the other hand, PAM-5 was less toxic than polymyxin B to Vero cells as reflected by the overall significantly higher RFUs from the former than the latter (*p* < 0.05) at the same range of concentrations.

### 3.10. Haemolytic Effect of PAM-5 to hRBCs

**Figure 6:**
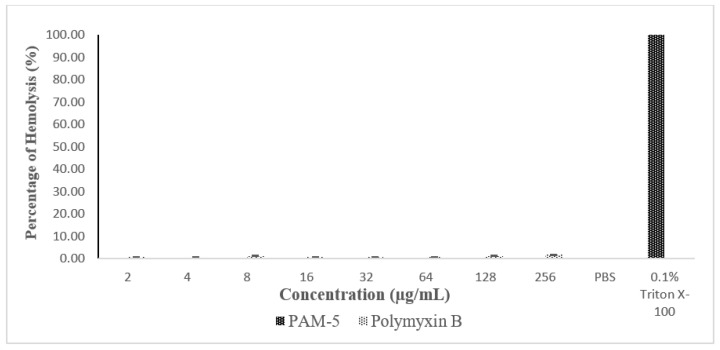
Percentage of haemolysis caused by peptide treatment in human red blood cells (hRBCs) based on haemolytic assay. hRBCs treated with PAM-5 at concentrations ranging from 2 µg/mL to 256 µg/mL showed almost undetectable haemolysis. The degree of haemolysis was even lower than RBCs treated with polymyxin B. hRBCs treated with 0.1% Triton X-100 and suspended in PBS served as the positive and negative control for haemolysis, respectively.

The haemolytic effect of PAM-5 was tested on hRBCs using an in vitro haemolytic assay. Based on Figure 6, PAM-5 did not produce measurable haemolytic effect on the RBCs as indicated by the significant differences in percentage of haemolysis between the peptide treatment and positive control (*p* < 0.05).

## 4. Discussion

The alarming state of global antibiotic resistance has called for exploration and development of novel antibacterial agents with distinct mechanism of actions from the conventional antibiotics against resistant bacteria [34]. Antibacterial peptides (ABPs) have been gaining considerable research attention due to their promising features that are able to overcome the limitations of conventional antibiotics. However, isolation of ABPs from natural resources could be tedious and the yields are usually uncertain, adding to the fact that many natural ABPs are toxic to mammalian cells [19,20,21]. These major drawbacks have prompted alternative approaches for ABP development that are able to customize peptides with potent antibacterial activity but low toxicity to human cells. One of these approaches is the combination of phage display selection and computer-assisted modification.

In this study, we used a 12-mer random phage-displayed peptide library to select for peptides that interact with the whole cell of *P. aeruginosa* in solution biopanning instead of specific purified epitopes of the bacteria. This biopanning strategy was used by many research groups to identify and design ABPs against many pathogenic bacteria, including *Haemophilus influenzae* [24], *Campylobacter jejuni* [25], *P. aeruginosa* [26], *S. aureus* [35] and *Listeria monocytogenes* [36]. As demonstrated by these studies, one of the advantages of whole cell biopanning is the selection of multiple peptide candidates that may have bound to various surface ligands of the bacteria, which provides researchers with the flexibility of identifying and selecting peptide/s with the most potent antibacterial effect for further development. On the other hand, whole-cell biopanning may also select a peptide or a motif within a peptide that binds to a conserved ligand that is present throughout the bacterial surface [37], where this binding may lead to antibacterial activity of the peptide.

Correspondingly, biopanning against the whole cells of *P. aeruginosa* in this study had consistently selected a peptide (5 out of 15) throughout the four rounds of affinity selection, namely Pa4 (KWHWKDKNALRM). Interestingly, this peptide contained a consensus motif Leu-Arg-Met (LRM) that was also found in a peptide with the second-highest selection frequency, Pa1 (GPVNKSSTILRM) (3 out of 15). This suggests that the motif “LRM” may have bound to a dominant multivalent ligand which may present extensively throughout the bacterial surface, contributing to the predominant selection of the two different peptides carrying the same motif during the whole-cell biopanning. With reference to the biochemical nature of this motif, it is believed that the two hydrophobic amino acids of this motif (L and M) may promote partitioning of the peptide into the hydrophobic membrane lipid bilayer of the bacteria, while the cationic amino acid (R) may further strengthen the binding between the motif and a possible anionic residue that is present within the vicinity of the bacterial ligand via electrostatic interaction. This speculation was supported by the analysis from the Antimicrobial Peptide Database (Peptide Calculation and Prediction) (https://aps.unmc.edu/helix, accessed on 15 April 2017), which suggested that Pa4 might form alpha helices and interact with the bacterial membrane, thus having a chance to be an antimicrobial peptide. Based on these considerations, Pa4 was chosen for further evaluation of its antibacterial potency.

Nevertheless, Pa4 might not be able to serve as a potent membrane-active ABP due to its relatively low cationicity (+3). It is well documented that peptide cationicity serves as an important factor to many ABPs that kill bacteria via membrane-disruptive or permeabilizing mechanisms, as this feature promotes initial peptide interaction to the anionic lipid head groups within the bacterial membrane [38,39]. Furthermore, a study by Jiang et al. (2009) [40] had demonstrated that a decrement in net positive charge below +4 may impair the antibacterial activity of an ABP. Most of the strong ABPs carry an average cationicity of > +6 [41,42,43,44], which is far more cationic than Pa4. Secondly, a thorough review on many ABPs revealed that almost half of the amino acids in the primary sequence of these peptides belonged to hydrophobic residues [45,46]. This indicates that the percentages of hydrophobicity of a potent ABP is approximately around 50%, which might anticipate the weaker antibacterial effect of Pa4 with a relatively lower hydrophobicity (41%). Therefore, Pa4 was subjected to rational modification by peptide elongation and amino acid substitution to enhance its cationicity and hydrophobicity ratio.

The computer-assisted modification as described above conferred the newly modified peptide, PAM-5 (KWKWRPLKRKLVLRM), with enhanced features of potent ABPs due to greater cationicity (+7) and hydrophobicity (47%). This was indicated by its potent bactericidal effect towards *P. aeruginosa* ATCC 27853, the target bacterium used for the affinity selection of its parental peptide (Pa4). Apart from this, PAM-5 was also found active against a panel of Gram-negative bacteria, including some multidrug-resistant clinical isolates at the MBCs that ranged from 4 µg/mL to 32 µg/mL. Interestingly, the bactericidal effect of PAM-5 was not compromised by a clinical isolate of carbapenem-resistant *K. pneumoniae*, which is commonly regarded as a superbug [47]. Clearly demonstrated by these findings, the non-bacterial-specific action by PAM-5 may render this peptide with the ability to inhibit or kill a broad spectrum of pathogenic bacteria, where a single antibacterial agent is able to kill bacteria from different families such as Pseudomonadaceae (*P. aeruginosa*), Enterobacteriaceae (*E. coli, K. pneumoniae, Salmonella* Typhi, *Shigella flexneri*) and Moraxellaceae (*A. baumannii, A. junii*). This might imply that PAM-5 could serve as a potential candidate for empiric treatment against bacterial infection in clinical settings where the etiological agent of infection is yet to be determined.

The broad spectrum of Gram-negative bacteria targeted by PAM-5 may be attributed to several possible reasons. Firstly, the biopanning process may have predominantly selected a peptide or short motif that is bound to a conserved ligand commonly expressed by many Gram-negative bacteria. Secondly, the enhanced cationicity of the newly modified peptide may promote stronger electrostatic interaction between the peptide and many anionic entities on Gram-negative bacterial membranes, such as phosphotidylglycerol (PG), phosphotidylserine (PS) and cardiolipin (CL) [48], while the increased peptide hydrophobicity may enable prolonged interfacial engagement between the two entities [49]. Corresponding to a few other studies [50,51,52], the higher molecular weight of elongated PAM-5 may produce higher lateral pressure on the bacterial membrane interface upon intercalation by the peptide, leading to local disruption to the membrane lipid. Moreover, certain minimum length of ABPs is essential for their amphipathicity and helical structure, which are common characteristics for many membrane-active ABPs to traverse the lipid bilayer of the bacterial membrane. Correspondingly, as predicted by the Antimicrobial Peptide Database (Peptide Calculator and Predictor), the addition of three amino acids to Pa4 resulted in increased helical structure and number of hydrophobic interfaces to the peptide, which might enhance the antibacterial effect of the newly designed PAM-5 as reported in this study.

Nevertheless, PAM-5 was generally less active against the Gram-positive bacteria selected for this study. Except for a clinical strain of *S. anginosus*, PAM-5 was less potent against *S. aureus* and *S. pyogenes* and not active against *E. faecalis*. This poor efficacy could be attributed to the barrier effects of thick peptidoglycan layers in Gram-positive bacteria, which might reduce the direct contact and accumulation of PAM-5 on the bacterial membrane. The similar ineffectiveness due to this barrier effect was also observed for several other membrane-active ABPs on Gram-positive bacteria [53,54,55].

The in vivo stability of ABPs remains as a major challenge to their clinical application. Once administered into the host body, ABPs might be subjected to different degrees of impairment due to proteolytic degradation or protein adsorption in serum, plasma, blood and other tissues [56,57,58]. In this study, PAM-5 was tested for its stability in human plasma via an ex vivo antibacterial assay, in which the peptide was incubated with the target bacteria suspended in human plasma instead of PBS. The stability of PAM-5 was evaluated by comparing the peptide MBC towards the target bacterium between the ex vivo and in vitro assay as reported earlier. In the presence of plasma, PAM-5 could only achieve complete killing of *P. aeruginosa* at the concentration of two-fold (16 µg/mL) the MBC of in vitro assay (8 µg/mL), suggesting the presence of certain inhibitory factors in the plasma that may interfere with the bactericidal activity of PAM-5 but yet to cause complete inactivation or degradation to the peptide. The slightly higher MBC of PAM-5 in the ex vivo assay indicated that this peptide was relatively stable in human plasma. According to Nguyen et al. (2010) [59], the number of tryptophan (W) and arginine (R) in an ABP is directly proportional to the peptide stability as these residues were found to reduce interaction between the peptide and proteolytic enzymes in the plasma. Since these two amino acids are also present in PAM-5 in considerable proportions (two W and three R), it is believed that PAM-5 is rendered with a certain degree of resistance towards the proteolytic enzymes in the plasma.

The ability of PAM-5 to kill several multidrug-resistant bacteria as reported in this study clearly indicated that the peptide may exert its bactericidal effect via mechanisms that are distinct from the conventional antibiotics, and these actions were not compromised by the common bacterial resistance mechanisms that are directed towards the latter. It is well established that many cationic and amphipathic ABPs kill their target bacteria via membrane-active mechanisms [27,31,32,60,61,62]. PAM-5 is a cationic peptide with moderate hydrophobicity, thus it is assumed to exert its bactericidal effect via the similar mechanism. To address this speculation, we first used scanning electron microscopy (SEM) to screen for morphological changes or damage to *P. aeruginosa* and *E. coli* after treatment with PAM-5. Clearly demonstrated from the SEM images, extensive surface disruption and shape deformity characterized by surface roughening and corrugation, blebbing formation and micelles were seen for PAM-5-treated *P. aeruginosa* and *E. coli* but not on untreated bacteria. These changes resembled the surface disruptions that were caused by several membrane-active ABPs from other studies [47,50,63,64,65] which possess the similar extent of cationicity and amphipathicity as PAM-5. As proposed by these studies, the two peptide features may allow these peptides to engage with multiple anionic sites of bacterial membranes in a non-ligand-selective manner. Further accumulation of ABPs may disrupt the integrity of the surface membrane, followed by weakening of the phospholipid bilayer and gradual collapse of the membrane into micelles or blebbings which were seen in this study.

Following the outer surface disruption, it is believed that PAM-5 could further permeabilize the bacterial cytoplasmic membrane if the peptide concentration is sufficient to reach the inner membrane. Thus, we carried out a SYTOX Green assay to verify this. SYTOX Green is a membrane-impermeable nucleic acid binder, but readily translocates permeabilized membrane into bacterial intracellular compartments and emits green fluorescence upon binding to nucleic acids (Invitrogen, Carlsbad, CA, USA). With reference to the results, PAM-5 was clearly indicated as a membrane-permeabilizing ABP when it was shown able to increase SYTOX Green uptake by the peptide-treated bacteria in a dose-dependent manner. It is believed that the increasing amount of PAM-5 could cause more extensive permeabilization of the bacterial cytoplasmic membrane, allowing more entry of SYTOX Green into the bacteria to bind with more nucleic acids and resulting in more fluorescent emission. However, the trend of fluorescent emission was inversely proportional to the concentration of peptide treatment at 128 µg/mL and 256 µg/mL, where a reducing fluorescent trend was observed at these two highest treatment concentrations. This unexpected finding might provide a plausible clue that PAM-5 might be able to bind to bacterial DNA. This speculation was eventually confirmed by electrophoretic mobility shift assay (EMSA), where PAM-5 was shown able to bind to bacterial genomic DNA from two species of bacteria and retard their migration during gel electrophoresis. Additionally, the peptide was also able to bind to plasmid DNA, suggesting its ability to interact with different forms of nucleic acids in bacteria. Interestingly, these DNA retardations typically occurred at high peptide concentrations (64 µg/mL to 256 µg/mL), which corresponded to the concentrations that resulted in decreasing fluorescent emission in the SYTOX Green assay as described earlier. The similar findings and assay verification were also reported by Taute et al. (2015) [66], where a defensin-derivative named Os was found to cause the similar trend of SYTOX Green fluorescent emission due to its ability to bind to bacterial DNA and displace the fluorescent probe from bacterial nucleic acids when it was present at high concentrations. It was suggested that ABP-DNA interaction is attributed to the positively charged amino acids such as lysine (K) and arginine (R) in the peptide, which might promote electrostatic interaction between the peptide and the negatively charged phosphate groups of the DNA backbone [67]. As these two residues made up almost half of PAM-5 (46.7%), strong electrostatic bonds were presumably formed between the peptide and bacterial DNA.

From the macroscopic view on the antibacterial actions of PAM-5, the findings on the surface disruption, inner membrane permeabilization as well as bacterial DNA binding may provide reasonable explanation for its bactericidal effect and rapid killing kinetics. Many membrane-active ABPs were found to exert rapid bactericidal effects [32,61,68,69,70] as these peptides may non-specifically interact with negatively charged entities that are present abundantly throughout the bacterial surface membrane. As the result of extensive surface damage, membrane depolarization, permeabilization and leakage that simultaneously occur throughout the entire bacterium, the bacterium may suffer from loss of intracellular components and die rapidly [46]. Secondly, binding of ABPs to bacterial genomic DNA may impair many genetic and proteomic activities such as DNA replication, DNA repair as well as gene expression for proteins that are essential for bacterial survival and proliferation [71], or even cause DNA breakage or chromosome segregation defects which are deadly to bacteria [72]. Although the detailed insights on these effects were not investigated for PAM-5 in this study, it is believed that the bactericidal effect of PAM-5 was partly attributed to DNA binding as reported earlier. Therefore, the extensive membrane damage and DNA binding by PAM-5 might contribute to its rapid killing kinetics, which suggests the peptide as a more potent antibacterial agent than many single target-specific, slow-acting antibiotics that are usually confronted by bacterial resistance via target modification.

Although a considerable large number of ABPs were documented in the Antimicrobial Database (https://aps.unmc.edu/database/anti, accessed on 25 October 2022), only a handful of them were approved for clinical application due to safety considerations. Similarly, despite exhibiting promising antibacterial effect, it is equally important to ensure the in vivo safety of PAM-5. To address this, we screened for the peptide’s cytotoxicity to Vero cells and haemolytic effect on human RBCs (hRBCs). Our data presented here clearly indicated that PAM-5 showed no remarkable cytotoxicity and haemolytic effects on these cells at all tested concentrations, encompassing certain high MBCs against several bacteria such as *Streptococcus pyogenes* (MBC: 64 µg/mL) and *Staphylococcus aureus* (128 µg/mL). Structural-functional studies have demonstrated that the balance between peptide hydrophobicity and hydrophilicity may critically influence the peptide selectivity between bacterial and mammalian cell membranes [73,74]. Studies have shown that a relatively low or moderate peptide hydrophobicity prevents its binding to zwitterionic mammalian cell membranes, thus precluding or reducing its toxicity to mammalian cells [75,76]. Conversely, increment of peptide hydrophobicity exceeding a certain optimal threshold may obfuscate this selectivity and promote its binding to mammalian cell membranes, thus resulting in peptide toxicity [76,77,78]. On the other hand, peptides’ cationicity has been associated with their electrostatic interaction with many negatively charged constituents such as lipopolysaccharide, phosphotidylglycerol (PG), phosphotidylserine (PS) and cardiolipin (CL) which are exclusively present on bacterial but not mammalian cell membranes [79,80,81,82]. In a study by Ma et al. (2014) [83], ABPs with net charges that range from +6 to +8 did not show remarkable sign of haemolysis, while the undesirable effect was only apparent if the peptide’s cationicity is reduced to below +6. The percentage of hydrophobicity of 46% and net positive charge of +7 for PAM-5 may void its toxicity effect towards Vero cells, as well as its haemolytic effect towards hRBCs. These could explain the insignificant toxicities and haemolytic effects of PAM-5 towards the cell line and hRBCs as reported in this study, which could be attributed to the rational modification of Pa4 of lower hydrophobicity (41%) and cationicity (+3) to PAM-5 with moderate hydrophobicity (46%) and higher cationicity (+7). However, further in vitro studies with other cell types and in vivo studies are needed to strengthen this notion.

## 5. Conclusions

Phage display selection accompanied by rational modification could yield ABPs with potent antibacterial effect. One product of this combined approach, PAM-5, was found to be a potent antibacterial peptide against a spectrum of Gram-negative pathogenic bacteria, including drug- and multidrug-resistant bacteria. PAM-5 stands a better chance as a more potent antibacterial agent as compared to many conventional antibiotics as it kills bacteria by multiple mechanisms of actions in a rapid manner. Finally, as a result of selection by bacterial ligands via biopanning and rational adjustment of the selected peptide, PAM-5 possesses relatively high selectivity by acting exclusively on bacteria but shows no obvious toxicity to mammalian cells. Hence, PAM-5 can be proposed for further evaluation in terms of peptide stability enhancement and pre-clinical and clinical studies to justify its potential role as an alternative antibacterial agent in clinical settings.

## Figures and Tables

**Table 1:** Sequences and characteristics of phage-displayed peptides selected from the fourth round of biopanning against *P. aeruginosa*.

Peptide	Sequence	Frequency of Selection	Net Charge	Percentage of Hydrophobicity
Pa1	GPVNKSSTILRM	3/15	+2	33%
Pa2	AHGNAALVARLK	1/15	+2	58%
Pa3	GLHTSATNLYLH	2/15	0	33%
Pa4	KWHWKDKNALRM	5/15	+3	41%
Pa5	GSLRPGTTNALV	2/15	+1	33%
Pa6	FGDLTRGQQRGP	1/15	+1	16%
Pa7	QGTVARLPIFWP	1/15	+1	50%

**Table 2:** MBCs of PAM-5 towards selected Gram-positive and Gram-negative bacteria screened in this study. The data were obtained from at least three independent rounds of assays.

Bacterial Strains	Relevant Features	MBC (µg/mL)
*P. aeruginosa* ATCC 27853	Reference strain	8
*P. aeruginosa* 12594264	(C.I.) LVX^R^ MXF^R^ DOR^R^ ETP^R^ MEM^R^ CAZ^R^ CRO^R^ FEP^R^ (MDR)	16
*E. coli* ATCC 25922	Reference strain	8
*E. coli* 1160702	C.I. AMC^I^ CFZ^R^ CXM^R^ CTX^R^ CRO^R^ GEN^R^ CIP^R^ (ESBL)	16
*A. baumannii* ATCC 19606	Reference strain	8
*A. junii* 1191828	C.I. CFZ^R^ CRO^R^ CAZ^R^	4
*K. pneumoniae* ATCC 13883	Reference Strain	32
*K. pneumoniae* 1208398	C.I. AMP^R^ AMC^R^ SAM^R^ TZP^R^ CFZ^R^ CXM^R^ FOX^R^ CTX^R^ CAZ^R^ CRO^R^ FEP^R^ ATM^R^ MEM^R^ AMK^R^ GEN^R^ CIP^R^ NIT^R^ (CRE)	8
*S.* Typhi 1238912	C.I. CAZ^R^ CTX^R^ GEN^R^	32
*S. flexneri* 1109563	C.I. CFX^R^ CFZ^R^ CXM^R^ AMK^R^ CIP^R^	32
*S. marcescens* 1191741	C.I. AMX^R^ CFZ^R^ CXM^R^ FOX^R^	>256
*S. aureus* ATCC 25923	Reference strain	128
*E. faecalis* ATCC 19433	Reference strain	>256
*S. pyogenes* ATCC 19615	Reference strain	64
*S. anginosus* 1360589	C.I.	4

C.I.: clinical isolate; CFZ^R^: resistance to cefazolin; LVX^R^: resistance to levofloxacin; MXF^R^: resistance to moxifloxacin; DOR^R^: resistance to doripenem; PB^R^: resistance to polymyxin B; ETP^R^: resistance to ertapenem, MEM^R^: resistance to meropenem; CAZ^R^: resistance to ceftazidime; CRO^R^: resistance to ceftriaxone; FEP^R^: resistance to cefepime; MDR: multi-drug resistance; AMC^I^: reduced susceptibility to amoxicillin/clavulanic acid; CXM^R^: resistance to cefuroxime; CTX^R^: resistance to cefotaxime; GEN^R^: resistance to gentamicin; CIP^R^: resistance to ciprofloxacin; ATM^R^: resistance to aztreonam; NIT^I^: reduced susceptibility to nitrofurantoin; AMX^R^: resistance to amoxicillin; AMP^R^: resistance to ampicillin; SAM^I^: reduced susceptibility to ampicillin/Sulbactam; TZP^R^: resistance to piperacillin/Tazobactam; FOX^R^: resistance to cefoxitin; AMK^R^: resistance to amikacin; ESBL: extended spectrum beta-lactamases; CRE: carbapenem-resistant Enterobacteriaceae.

## Data Availability

The data reported in this study are available in the article.
